# The role of NAD^+^ metabolic reprogramming in colorectal cancer chemoresistance: mechanistic insights, clinical translation challenges and opportunities

**DOI:** 10.3389/fonc.2026.1784749

**Published:** 2026-05-28

**Authors:** Zhennan Ma, Xiuqing Gong, Fuquan Liu

**Affiliations:** 1 Department of Anorectal Surgery, Dalian University Affiliated Xinhua Hospital, Dalian, China; 2 Obstetrical Ward 1, Dalian Women and Children Medical Center, Dalian, China

**Keywords:** chemoresistance, colorectal cancer, metabolic reprogramming, NAD+, targeted intervention

## Abstract

As a pivotal hub of cellular metabolism, NAD^+^ metabolic reprogramming exerts a core role in colorectal cancer chemoresistance by regulating energy metabolism, DNA repair, and the immune microenvironment. The dysregulation of synthetic and catabolic pathways mediated by key molecules such as NAMPT, SIRT1, and PARP constitutes a crucial mechanism underlying chemoresistance development. Targeted intervention strategies against NAD^+^ metabolism, including precursor supplementation, inhibitor administration, and combination therapy, have exhibited remarkable anti-cancer potential and represent promising translational strategies for reversing chemoresistance. However, clinical translation of these strategies is severely impeded by tumor metabolic heterogeneity, the lack of dynamic NAD^+^ monitoring technologies and insufficient tissue specificity of targeted drugs. By leveraging emerging techniques including multi-omics integration, organoid models, nano-delivery systems, and dynamic imaging, in-depth dissection of metabolic heterogeneity and development of personalized intervention regimens can provide novel and effective avenues to overcome the predicament of colorectal cancer chemoresistance, which holds important translational research value and clinical application significance.

## 1 Introduction

Colorectal cancer (CRC) is a highly prevalent and lethal malignancy worldwide, with chemoresistance representing a major bottleneck in clinical treatment. Although standard chemotherapy regimens based on 5-fluorouracil and oxaliplatin are widely administered, numerous patients develop intrinsic or acquired drug resistance, resulting in disease progression and reduced survival (1). The mechanisms of chemoresistance are multifactorial, including drug efflux, enhanced DNA damage repair and other biological processes (2–4). Tumor metabolic reprogramming acts as a critical driver of drug resistance, among which the aberrant activation of the NAD^+^ metabolic network has emerged as a research hotspot.

As an indispensable coenzyme and signaling molecule, NAD^+^ is extensively involved in fundamental physiological processes, including energy metabolism, redox homeostasis, epigenetic regulation, and DNA repair (5, 6). Serving as an essential substrate for Sirtuin deacetylases and PARP family poly(ADP-ribose) polymerases, NAD^+^ plays a central role in maintaining genomic stability, regulating mitochondrial function, and mediating cellular stress responses. Emerging evidence indicates that dynamic alterations in intracellular NAD^+^ levels directly determine cell fate. Under oxidative stress, SIRT3 sustains mitochondrial homeostasis and restrains reactive oxygen species (ROS) accumulation by modulating the activities of SOD2 and PGC-1α, thereby endowing colorectal cancer cells with potent chemoresistance (7). In the context of DNA damage, excessive PARP activation drastically depletes cellular NAD^+^ pools, indirectly impairs SIRT1 function, and further modulates downstream glycolytic pathways and cell survival (8). Such coupled metabolic and signaling mechanisms render NAD^+^ homeostasis a critical hub linking metabolic adaptation to therapeutic response in CRC.

In colorectal cancer, NAD^+^ metabolic reprogramming facilitates chemoresistance through synergistic multi-layered mechanisms. First, the upregulation of key rate-limiting enzymes in NAD^+^ synthetic pathways, such as NAMPT, sustains elevated intracellular NAD^+^ levels to support PARP-mediated DNA damage repair, enabling tumor cells to evade chemotherapy-induced genotoxic cell death (9). Second, hyperactivated SIRT1 inhibits apoptosis and accelerates cell cycle progression via deacetylation-mediated regulation of transcription factors including NF-κB and p53 (10, 11). Moreover, NAD^+^ metabolism profoundly modulates the functional state of immune cells within the tumor microenvironment. CD8^+^ T cells rely on the kynurenine pathway for autonomous NAD^+^ synthesis to sustain anti-tumor immunity, whereas tumor-associated B cells reinforce their immunosuppressive phenotypes through LARS2-driven mitochondrial NAD^+^ regeneration (12, 13). Accordingly, targeting NAD^+^ metabolism can directly abrogate the survival advantages of malignant cells and remodel the immunosuppressive microenvironment, thereby eliciting synergistic anti-tumor effects (14).

This review systematically summarizes the molecular mechanisms by which NAD^+^ metabolic reprogramming drives chemoresistance in colorectal cancer, and evaluates the translational potential of current intervention strategies, including NAD^+^ precursor supplementation, key metabolic enzyme inhibitors, and nanodelivery systems. Additionally, it highlights major bottlenecks hindering clinical translation, such as tumor metabolic heterogeneity, the lack of dynamic monitoring technologies, and insufficient tissue specificity of targeted agents. The integration of multi-omics analysis, patient-derived organoid models, and advanced *in vivo* imaging techniques is expected to enable precise stratification of chemoresistant subtypes and individualized metabolic intervention. This work aims to provide in-depth theoretical insights and clinically viable novel strategies to overcome the therapeutic bottlenecks of colorectal cancer.

## 2 Molecular mechanisms of NAD^+^ metabolic imbalance

### 2.1 NAD^+^ biosynthetic and catabolic pathways

Mammalian cells generate NAD^+^ via three biosynthetic pathways, with distinct relevance in CRC (15). The *de novo* pathway, initiating with tryptophan, is minimally active in CRC due to low QPRT activity, failing to meet tumor metabolic demands (9).

The Preiss-Handler pathway, relying on exogenous nicotinic acid and NAPRT, is also limited in CRC as NAPRT is frequently silenced by promoter methylation (16). In contrast, the salvage pathway predominates in CRC, using nicotinamide (NAM) as substrate—NAMPT, the rate-limiting enzyme, is often overexpressed in CRC, sustaining NAD^+^ pools for DNA repair and cancer stem cell properties via SIRT1/PARP activation, directly linking to chemoresistance (17). NMNAT2, the major CRC cytosolic nicotinamide mononucleotide adenylyltransferases (NMNAT) isoform, correlates with advanced TNM stage and p53 dysregulation, supporting genomic instability under chemotherapy (18, 19).

NAD^+^ catabolism in CRC is mediated by PARPs, SIRTs, and CD38. PARP1 is hyperactivated by oxaliplatin-induced DNA damage in resistant CRC cells, depleting NAD^+^ and suppressing SIRT1, which enhances glycolysis (via elevated PKM2/LDHA) and sustains survival (8, 20); PARP inhibition restores NAD^+^ and resensitizes cells (8). SIRTs are a family of NAD^+^-dependent deacetylases that participate in the regulation of metabolism, stress responses, and the expression of aging-related genes, with their activity directly modulated by intracellular NAD^+^ concentrations (21). SIRT3 maintains mitochondrial integrity via SOD2/PGC-1α, reducing chemotherapy-induced mtROS and protecting tumor cells (7).

**Figure 1:**
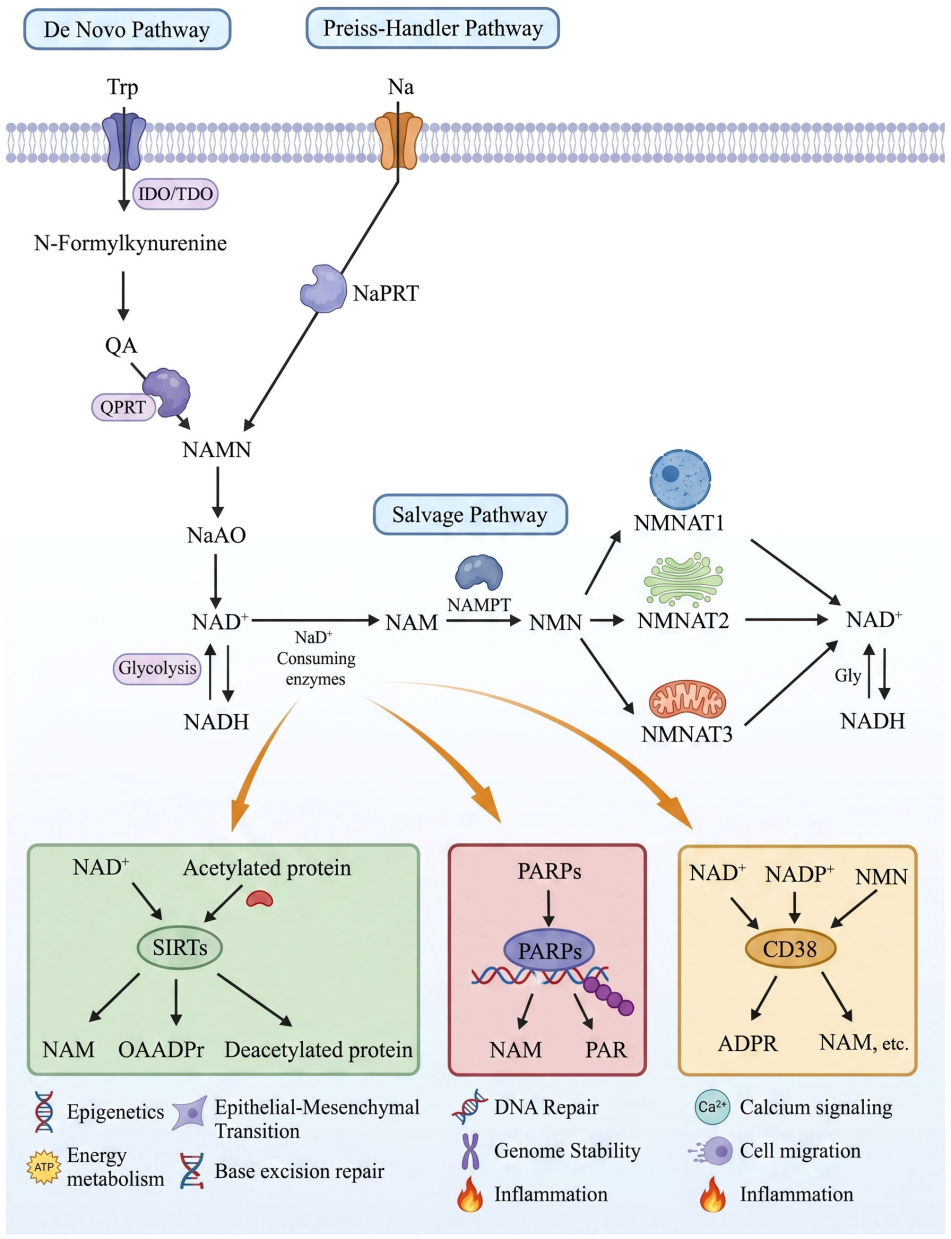
NAD^+^ biosynthetic and catabolic pathways in mammalian cells. Mammalian cells produce NAD^+^ via three pathways: the *de novo* pathway, the Preiss-Handler pathway, and the salvage pathway. NAD^+^ catabolism is mediated by PARPs, SIRTs, and CD38, which not only regulate NAD^+^ levels but also generate bioactive metabolites. Abbreviations: Trp, tryptophan; IDO/TDO, indoleamine 2,3-dioxygenase/tryptophan 2,3-dioxygenase; QA, quinolinic acid; QPRT, quinolinic acid phosphoribosyltransferase; NaMN, nicotinic acid mononucleotide; NaAO, nicotinic acid adenine dinucleotide synthetase; NaAD, nicotinic acid adenine dinucleotide; NA, nicotinic acid; NaPRT, nicotinic acid phosphoribosyltransferase; NAM, nicotinamide; NAMPT, nicotinamide phosphoribosyltransferase; NMN, nicotinamide mononucleotide; NMNAT1-3, nicotinamide mononucleotide adenylyltransferases 1-3; PARPs, poly(ADP-ribose) polymerases; CD38, cluster of differentiation 38; SIRTs, sirtuins.

CD38 hydrolyzes NAD^+^ and mediates metabolite interconversion, with potential roles in CRC tumor microenvironment remodeling that warrant further exploration (22). Disruption of NAD^+^ biosynthesis-catabolism balance depletes intracellular NAD^+^, impairing energy metabolism, epigenetics, and redox homeostasis—laying the foundation for CRC chemoresistance (23). **Figure 1** clearly illustrates the synthesis and catabolic mechanisms of NAD^+^, visually presenting the three aforementioned synthetic pathways and the metabolic networks mediated by three types of catabolic enzymes.

### 2.2 Drivers of NAD^+^ metabolic imbalance

NAD^+^ metabolic imbalance arises from both tumor-intrinsic metabolic reprogramming and microenvironmental stresses including aging, chronic inflammation, DNA damage, and mitochondrial dysfunction, which collectively lower tissue NAD^+^ levels and represent a common metabolic hallmark of cancer. A key distinction exists between systemic NAD^+^ decline and tumor-specific rewiring: systemic NAD^+^ depletion impairs normal tissue function, while cancer cells maintain high local NAD^+^ flux via upregulating salvage enzymes (e.g., NAMPT) or downregulating consumers (e.g., CD38), supporting proliferation, DNA repair, immune escape, and drug resistance. Differentiating systemic and tumor NAD^+^ status is critical for targeted therapy (24, 25).

Aging-related NAD^+^ reduction is driven by sustained PARP activation, inflammageing-induced CD38 overexpression, and impaired NAMPT-mediated salvage (26). CD38 is a major NAD^+^ consumer in immune and adipose tissues with aging (24). Chronic inflammation also elevates CD38, causing NAD^+^ depletion, mitochondrial dysfunction, and immune exhaustion in T cells (27). In the tumor microenvironment, tumor-associated macrophages highly express CD38, lowering local NAD^+^ and generating adenosine to promote immune evasion (23).

In CRC, chronic inflammation—a core driver of tumorigenesis—exacerbates NAD^+^ imbalance by inducing IL-6, TNF-α, and TGF-β, which upregulate CD38 in tumor-associated macrophages and stromal cells to accelerate NAD^+^ hydrolysis (28). This is especially evident in colitis-associated CRC, where persistent mucosal inflammation causes immune exhaustion and NAD^+^ depletion, fostering an immunosuppressive microenvironment (29).

Cumulative DNA damage is another major driver. Chemotherapy or replication stress chronically activates PARPs, consuming excessive NAD^+^. Cancer cells often counteract this by upregulating NAMPT to sustain NAD^+^ homeostasis and survival under therapeutic stress (30). Notably, CRC cells often buffer such NAD^+^ consumption by upregulating NAMPT expression to maintain intracellular NAD^+^ homeostasis and support survival under therapeutic stress (31). In CRC, the NAMPT inhibitor FK866 reduces NAD^+^ in cancer-associated fibroblasts (CAFs), attenuating their pro-metastatic function and emphasizing NAD^+^ metabolism in stromal–tumor crosstalk (31).

Mitochondrial dysfunction worsens NAD^+^ imbalance by impairing NAD^+^ regeneration and increasing oxidative stress, forming a vicious cycle (32). In CRC, this vicious cycle is particularly pronounced: mitochondrial dysfunction not only blocks NAD^+^ recycling but also amplifies oxidative stress, further promoting metabolic reprogramming and chemoresistance (33). SIRT inhibitors (e.g., MHY2245, MHY2255) in HCT116 cells increase p53 acetylation and DNA damage, inducing cell cycle arrest and apoptosis via the JNK/p53 pathway, highlighting the role of NAD^+^-dependent deacetylases in CRC survival (10, 34).

CRC cells rewire NAD^+^ metabolism via NAMPT upregulation or CD38 downregulation to drive proliferation, survival, and chemoresistance. Mitochondrial SIRT3 mediates chemoresistance by enhancing antioxidant capacity via SOD2 and PGC-1α, reducing mtROS and protecting CRC cells from apoptosis (7). High SIRT3 correlates with poor survival in CRC patients, supporting its prognostic and therapeutic value (7). Targeting NAMPT combined with STAT3 inhibition synergistically suppresses CRC via ferroptosis induction and immune microenvironment remodeling, offering a strategy to overcome chemoresistance (14). These multifaceted drivers and cancer-type-specific alterations of NAD^+^ metabolic imbalance are systematically summarized in **Supplementary Table 1**.

## 3 Molecular mechanisms underlying CRC chemoresistance mediated by NAD^+^ metabolism

### 3.1 Clinical hazards of chemoresistance in CRC and research advances in metabolic reprogramming

CRC ranks as the second leading cause of cancer-related deaths worldwide, and its high recurrence rate and therapeutic resistance have severely limited the survival benefits of patients (35). Despite continuous advances in surgical resection, chemotherapy, targeted therapy, and immunotherapy, up to 90% of patients with metastatic CRC eventually develop chemoresistance, leading to disease progression and treatment failure (36). The mechanisms of chemoresistance are complex and multifactorial, involving genetic mutations, epigenetic regulation, activation of cancer stem cell properties, and remodeling of the tumor microenvironment (35, 37). In recent years, metabolic reprogramming, as a core strategy for cancer cells to adapt to therapeutic stress, has been widely recognized as a crucial biological basis driving CRC chemoresistance.

Glucose metabolic reprogramming—particularly the Warburg effect—has been repeatedly identified as a core mechanism promoting CRC cell proliferation, invasion, and resistance to first-line chemotherapeutic agents such as 5-fluorouracil (5-FU) and oxaliplatin. It is noteworthy that although 5-FU and oxaliplatin are often mentioned together as first-line chemotherapeutic agents for CRC, they exhibit fundamental differences in their mechanisms of action and interactions with the NAD^+^ metabolic axis. Oxaliplatin primarily activates PARP-1 by inducing DNA double-strand breaks, thereby depleting NAD^+^ and inhibiting SIRT1, which drives glycolysis-dependent drug resistance (8). Unlike 5-FU, an antimetabolite that primarily disrupts RNA and DNA synthesis without inducing extensive direct DNA strand breaks, its relationship with the PARP−SIRT1 axis warrants more careful assessment. Nevertheless, emerging evidence supports a functional role for this axis in 5−FU resistance. GPR15 has been shown to facilitate cytoplasmic NAD^+^ accumulation by suppressing PARP4, which in turn enhances central carbon metabolism through mitochondrial translocation, thereby priming tumor cells for 5−FU sensitivity. Furthermore, the PARP inhibitor rucaparib acts synergistically with 5−FU to exert potent antitumor activity in both organoid and xenograft models (38). Separately, FTO modulates G6PD and PARP1 expression via demethylation, altering NADPH homeostasis and oxidative stress responses during 5−FU exposure, which indirectly contributes to the establishment of a drug−resistant phenotype (39).

Key glycolytic enzymes including HK, PKM2, and LDHA not only support rapid ATP production and biosynthesis but also confer survival advantages to tumor cells by regulating redox homeostasis (40, 41). Notably, the enzymatic activity of several glycolytic regulators is directly modulated by NAD^+^-dependent deacetylases like SIRT1 and SIRT6, linking glycolytic flux to the cellular NAD^+^ pool and underscoring how Warburg-driven chemoresistance may be pharmacologically targetable via NAD^+^ modulation (9, 42).

Meanwhile, mitochondrial metabolic remodeling has also attracted increasing attention: studies have shown that the dysregulation of mitochondrial fusion/fission dynamics can enhance oxidative phosphorylation (OXPHOS) capacity, induce stemness phenotypes, and mediate oxaliplatin resistance. This OXPHOS adaptation often coincides with elevated mitochondrial NAD^+^ levels and increased SIRT3 activity, which deacetylates and activates antioxidant enzymes such as SOD2 and metabolic regulators like PGC-1α—thereby buffering oxidative stress induced by chemotherapy and reinforcing chemoresistance (7).

In addition, lipid metabolism (e.g., DHCR7-mediated cholesterol synthesis), amino acid metabolism (e.g., ACSL5 sustaining glycolysis and OXPHOS under glutamine deprivation), and even microbial metabolites (e.g., Fusobacterium nucleatum inducing myeloid-derived suppressor cell recruitment and epithelial-mesenchymal transition, or EMT) have all been confirmed to participate in the establishment of a chemoresistant microenvironment. Recent work reveals that cholesterol biosynthesis, while seemingly distant from NAD^+^, can suppress cGAS-STING–mediated immunogenic cell death—a process whose efficacy is known to be modulated by PARP activity and thus sensitive to NAD^+^ availability (43). Similarly, ACSL5-driven fatty acid oxidation generates NADPH, a reducing equivalent whose regeneration relies indirectly on NAD^+^-dependent pathways such as the malate-aspartate shuttle and mitochondrial transhydrogenase activity; depletion of NAD^+^ therefore compromises redox buffering capacity downstream of ACSL5, sensitizing cells to oxidative damage from chemotherapy (44, 45). Even microbial influences like F. nucleatum, which remodels the immune microenvironment, may exert indirect effects on NAD^+^ metabolism by inducing chronic inflammation—a state known to upregulate NAMPT and accelerate NAD^+^ turnover, potentially creating a metabolic vulnerability exploitable by NAD^+^-lowering agents (9, 42).

NAD^+^ acts not only as a critical cofactor for glycolysis, the TCA cycle, and OXPHOS but also as an essential substrate for the PARP family and SIRT deacetylases. Latest research has demonstrated that oxaliplatin-induced DNA damage can excessively activate PARP-1, resulting in massive depletion of the intracellular NAD^+^ pool. This process further inhibits SIRT1 activity, thereby abrogating its negative regulation of key glycolytic enzymes PKM2 and LDHA, ultimately driving metabolic reprogramming and the establishment of a chemoresistant phenotype (8, 20). Conversely, restoring SIRT1 expression or inhibiting glycolysis can effectively reverse chemoresistance.

### 3.2 Expression and regulatory mechanisms of key molecules in NAD^+^ biosynthetic and catabolic pathways underlying CRC chemoresistance

NAD^+^ metabolic reprogramming is critical for CRC cells to resist chemotherapy. In the biosynthetic pathway, NAMPT, the rate-limiting enzyme of the salvage pathway, has been identified as a metabolic initiating hub in plasma proteomic studies of patients with early-stage CRC. Its high expression is not only significantly correlated with resistance to 5-FU/FOLFOX chemotherapy but also promotes EMT via activating the PI3K/Akt signaling pathway, thereby enhancing tumor invasiveness and therapeutic resistance (46). Meanwhile, in CRC models, NAMPT overexpression further consolidates the EMT phenotype and treatment resistance by enhancing PARP/SIRT1-mediated DNA repair and stemness maintenance programs (17).

Apart from NAMPT, NAPRT also exerts indispensable pro-resistance effects in CRC. Immunohistochemical analysis of 261 CRC clinical specimens demonstrated that high expression of either NAMPT or NAPRT serves as an independent risk factor for shortened overall survival and disease-free survival. Their upregulation is closely associated with vascular invasion, deep tumor infiltration and advanced TNM stage. Notably, patients with concurrent high expression of both molecules exhibit the worst clinical outcomes, suggesting that NAMPT and NAPRT synergistically sustain intracellular NAD^+^ pools under chemotherapeutic stress (47). In addition, these two molecules possess distinct regulatory patterns: NAMPT is predominantly modulated by microRNAs, whereas NAPRT is more susceptible to genomic aberrations including gene amplification, mutation and DNA methylation. Such divergent regulatory mechanisms further contribute to the heterogeneous chemoresistance phenotypes in CRC (47).

Preclinical models have shown that FK866, a NAMPT inhibitor, can effectively deplete the intracellular NAD^+^ pool and reverse the sensitivity of PARG inhibitor-resistant cells to radiotherapy and chemotherapeutic agents, indicating that NAMPT acts not only as a metabolic node but also as a key regulatory factor in the chemoresistance network (48). Oxaliplatin-induced PARP-1 overactivation drives NAD^+^ depletion and suppresses SIRT activity in chemoresistant CRC (23, 46).

Latest research based on nanotechnology has further revealed that oxygen vacancy-engineered bimetallic nanozymes can irreversibly degrade NAD^+^/NADH, disrupt the electron transport chain, and inhibit the glutathione S-transferase P1 (GSTP1) pathway, thereby exerting a synergistic effect to enhance the efficacy of oxaliplatin in chemoresistant CRC (49).

### 3.3 Multilevel mechanisms of chemoresistance mediated by core NAD^+^-dependent enzymes in CRC

As major effectors of NAD^+^, the SIRT family of deacetylases exhibit highly context-dependent functional heterogeneity in CRC chemoresistance. SIRT1 downregulation is a hallmark event in oxaliplatin-resistant cells, a mechanism stemming from NAD^+^ depletion induced by excessive PARP activation. This depletion further inhibits SIRT1-mediated deacetylation of key glycolytic enzymes PKM2 and LDHA, leading to enhanced Warburg effect and energy metabolism remodeling (8). Restoring SIRT1 expression or administering SIRT1 agonists not only reverses the glycolytic phenotype but also enhances mitochondrial oxidative phosphorylation via a PGC1α-dependent mechanism, endowing tumor cells with metabolic flexibility and survival advantages under chemotherapeutic stress (50). In addition, SIRT1 is also involved in regulating the DNA damage response: FOXQ1 promotes p53 deacetylation by upregulating SIRT1 expression, thereby inhibiting apoptotic programs and augmenting resistance to platinum-based drugs (51). This dual role (metabolic regulator vs. apoptosis inhibitor) is not contradictory but reflects adaptive strategies to different tumor microenvironment stresses: addressing energy crises and evading drug-induced apoptosis.

Notably, both clinical samples and cell models have shown that SIRT1 is universally overexpressed in advanced CRC and metastases, and is significantly associated with poor prognosis (52), suggesting that its pro-survival and anti-apoptotic functions may predominate in most advanced tumor contexts. Furthermore, small-molecule inhibitors targeting SIRT1 (e.g., MHY2251) can effectively induce JNK/p53 pathway-mediated apoptosis in p53-wildtype CRC cells (10), further confirming its critical role as an apoptotic gatekeeper.

Therefore, when developing therapeutic strategies, it is necessary to accurately distinguish the “timing” and “targeting” of SIRT1 intervention based on the tumor’s metabolic state and p53 functional integrity: SIRT1 activators may be considered in glycolysis-dominated chemoresistant scenarios to restore oxidative phosphorylation balance (20), while SIRT1 inhibition should be prioritized in cases with intact p53 function and suppressed apoptosis to reactivate apoptotic programs (53).

**Figure 2:**
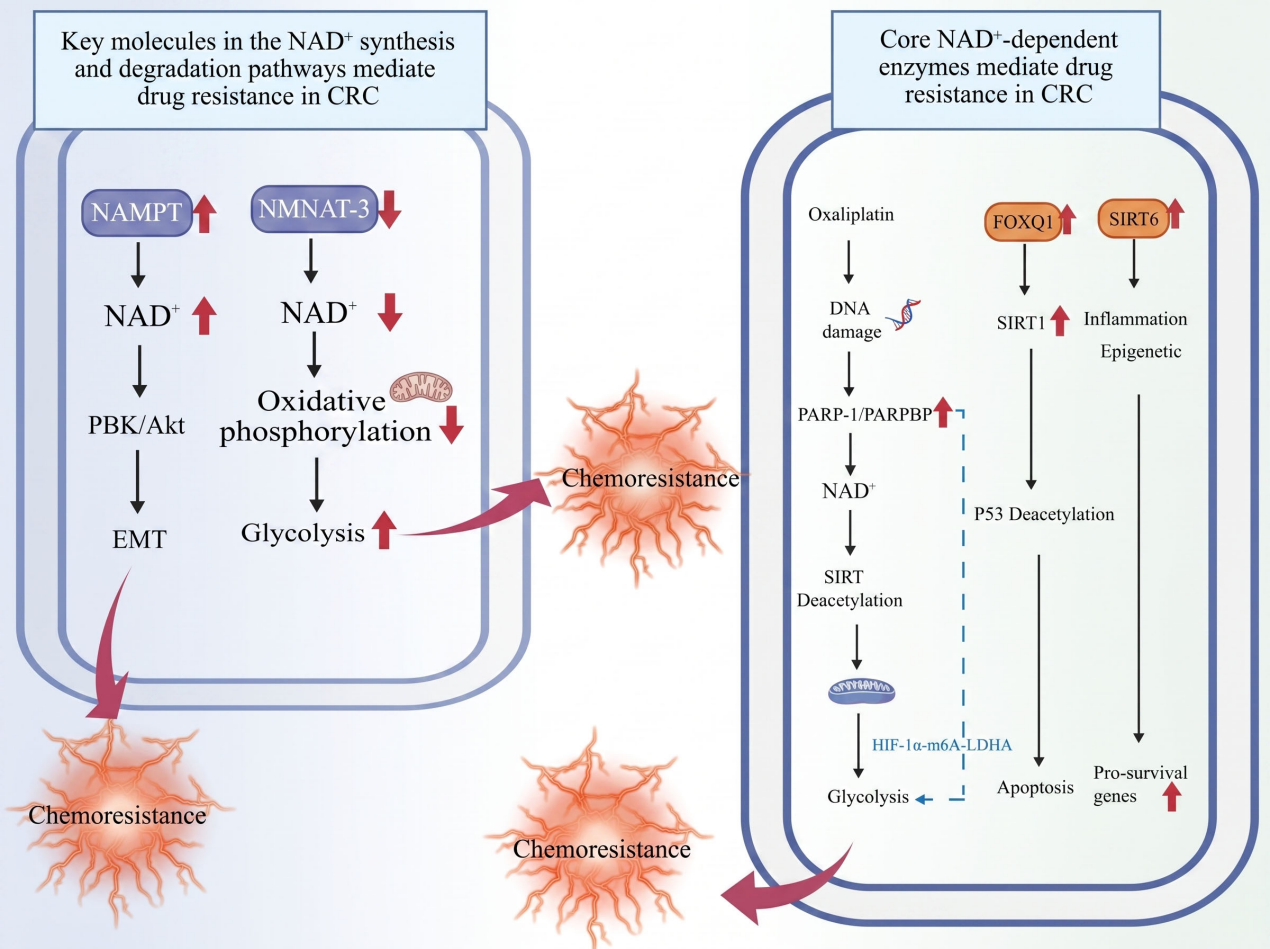
Mechanisms of NAD^+^ metabolism-mediated chemoresistance in CRC. This illustration delineates the dual regulatory axes through which dysregulated NAD^+^ metabolism drives chemoresistance in CRC. On the left, key molecules in NAD^+^ synthesis and degradation pathways exert their effects: upregulated NAMPT elevates intracellular NAD^+^ levels and activates the PI3K/Akt pathway to promote EMT, while downregulated mitochondrial NMNAT-3 reduces NAD^+^ pools and shifts cellular metabolism toward glycolysis. Oxaliplatin-induced DNA damage triggers overexpression of PARP-1/PARPBP, which depletes NAD^+^ and suppresses SIRT family activity. On the right, core NAD^+^-dependent enzymes mediate resistance through multi-layered mechanisms: upregulated FOXQ1 enhances SIRT1 expression to promote p53 deacetylation and inhibit apoptosis, and high SIRT6 drives resistance via inflammatory and epigenetic regulation. PARP-1 overactivation further stabilizes the HIF-1α-m6A-LDHA axis to enhance glycolysis, with all these alterations converging to induce chemoresistance in CRC cells.

Beyond SIRTs, the PARP family is also deeply implicated in chemoresistance development. Excessive PARP1 activation not only depletes intracellular NAD^+^ pools but also promotes glycolysis by stabilizing the HIF-1α-m6A-LDHA axis, directly conferring 5-FU resistance (54). Collectively, these findings indicate that NAD^+^-dependent enzymes integrate multiple mechanisms including metabolic reprogramming, epigenetic regulation, stemness maintenance, and inflammatory microenvironment remodeling to form a complex regulatory network underlying CRC chemoresistance. To achieve precise therapeutic breakthroughs, interventions targeting these enzymes need to take into account their enzymatic activity, subcellular localization, and crosstalk with upstream and downstream signaling pathways. As illustrated in **Figure 2**, the mechanistic roles of NAD^+^ metabolic pathways in CRC chemoresistance are systematically depicted.

## 4 Strategies and challenges of targeting NAD^+^ metabolism to intervene in CRC chemoresistance

### 4.1 Bidirectional effects of NAD^+^ precursor supplementation and inhibitor administration

In CRC, NAD^+^ metabolic reprogramming has become a pivotal driver of chemotherapy resistance. The core mechanism relies on the functional regulation of NAD^+^-dependent enzymes, especially sirtuin family proteins. Studies have shown that DNA damage induces excessive PARP activation in oxaliplatin-resistant CRC cells, which causes massive intracellular NAD^+^ consumption and further suppresses the activity of the NAD^+^-dependent deacetylase SIRT1 (8).

Downregulation of SIRT1 expression increases the levels of key glycolytic enzymes PKM2 and LDHA, strengthens glycolytic flux, grants tumor cells superior metabolic capacity, and ultimately promotes the formation of drug-resistant phenotypes (20). This evidence confirms a sequential regulatory chain, including PARP activation, intracellular NAD^+^ depletion, SIRT1 activity suppression, glycolysis enhancement, and the final development of chemoresistance.

Therefore, restoring SIRT1 function or supplementing NAD^+^ precursors is regarded as a promising approach to reverse tumor drug resistance. Relevant experiments have verified that exogenous upregulation of SIRT1 can efficiently restore the sensitivity of CRC cells to oxaliplatin and inhibit glycolysis. In contrast, the intervention of the SIRT1 inhibitor Salermide aggravates chemoresistance and elevates glycolytic levels.

Targeting the rate-limiting enzymes of NAD^+^ synthesis such as NAMPT also possesses prominent therapeutic value. As a classic NAMPT inhibitor, FK866 blocks the NAD^+^ salvage synthesis pathway, sharply reduces intracellular NAD^+^ content, triggers energy metabolism collapse, and leads to tumor cell death (55).

However, NAMPT inhibition still has limitations. Despite its favorable efficacy in some CRC models, long-term treatment tends to induce adaptive drug resistance through the activation of alternative metabolic pathways and the upregulation of DNA repair-related genes (56).

In addition to SIRT1, SIRT3 and SIRT6 also participate in the modulation of CRC chemoresistance. SIRT3 maintains cellular redox homeostasis by regulating the mitochondrial antioxidant enzyme SOD2 and the coactivator PGC-1α, and its high expression is closely linked to poor patient prognosis (7). SIRT6 exerts pro-apoptotic and chemosensitizing effects, and its low expression correlates with CRC progression and 5-FU resistance (57).

In summary, interventions targeting NAD^+^ metabolism present obvious bidirectional regulatory characteristics. Therapeutic strategies can inhibit specific enzymes such as PARP and NAMPT to reduce intracellular NAD^+^ content and weaken tumor cell survival. Meanwhile, activating protective sirtuin members including SIRT1 and SIRT6 can restore metabolic homeostasis and reverse chemotherapy resistance. Such mechanistic complexity requires accurate judgment of tumor metabolic characteristics, so as to avoid indiscriminate and unified therapeutic intervention strategies. **Table 1** summarizes representative strategies targeting NAD^+^ metabolism to reverse colorectal cancer chemoresistance.

**Table 1:** NAD+ metabolism in CRC chemotherapy resistance.

Intervention target	Intervention method	Combined chemotherapeutic drugs	Research model	Main effects	References
SIRT1	Exogenous overexpression or activation	Oxaliplatin	HCT116, HT29 cell lines and xenograft models	Restoring SIRT1 expression reverses oxaliplatin resistance, inhibits glycolysis, and enhances chemosensitivity.	(8, 20)
NAMPT	Inhibitor FK866	5-FU, cisplatin	HCT116 parental cells and drug-resistant strain HCT116R	Depletes NAD^+^ to induce energy crisis; drug-resistant strains become more sensitive to 5-FU/cisplatin.	(17, 56)
NAD^+^/Electron Transport Chain	Oxygen vacancy-rich bimetallic nanozyme (CuMnOx-V@Oxa@SP)	Oxaliplatin	Drug-resistant CRC cells and mouse models	Degrades NAD^+^/NADH, disrupts the electron transport chain, inhibits GSTP1, amplifies oxidative stress, and restores chemosensitivity.	(49)
Rev1-Rev7 complex	Inhibitor JH-RE-06	Mitomycin C (MMC)	HCT116 cell line	Inhibits translesion synthesis repair, alters the NAD^+^/NADH ratio, accumulates ROS, and enhances the cytotoxicity of MMC.	(58)
LARS2/SIRT1 axis	Lars2 gene knockout or leucine restriction	No specific chemotherapeutic drugs (immunomodulation as the main focus)	Mouse CRC models and human CRC tissues	Inhibits mitochondrial NAD^+^ regeneration and oxidative metabolism in regulatory B cells, weakens immune evasion, and improves anti-tumor immune responses.	(13)

### 4.2 Preclinical evidence for combination therapies to overcome chemoresistance barriers

Monotherapies targeting NAD^+^ metabolism often fail to achieve sustained efficacy due to compensatory mechanisms and toxic side effects. Accordingly, combination strategies have emerged as a core approach to overcome the bottleneck of chemotherapy resistance in CRC. A growing body of preclinical studies have verified the synergistic effects between NAD^+^ metabolism modulators and conventional chemotherapeutic agents. For instance, in oxaliplatin-resistant CRC models, restoration of SIRT1 expression exerts intrinsic anti-tumor activity and markedly potentiates the cytotoxicity of oxaliplatin. Meanwhile, combined administration with shikonin, a glycolysis inhibitor targeting PKM2, effectively reverses the drug-resistant phenotype induced by SIRT1 suppression, which validates the feasibility of dual intervention through SIRT1 activation and glycolysis inhibition (8, 20).

In another innovative study, an oxygen vacancy-engineered bimetallic nanozyme (CuMnOx-V@Oxa@SP) was constructed. This nano-system enables targeted delivery of loaded oxaliplatin, and its enzyme-mimicking activity can irreversibly degrade intracellular NAD^+^ and NADH. Such disruption impairs the electron transport chain and blocks the glutathione S-transferase P1 (GSTP1) pathway, thereby synergistically amplifying oxidative stress and disabling the antioxidant defense of tumor cells. Ultimately, this combinatorial approach prominently restores the chemosensitivity of drug-resistant CRC cells (49).

In addition, targeting the DNA repair pathway exhibits promising synergistic potential with NAD^+^ metabolic intervention. JH-RE-06, a selective inhibitor of the Rev1-Rev7 complex, enhances the cytotoxicity of mitomycin C (MMC) in HCT116 CRC cells. Mechanistically, this agent interferes with translesion synthesis (TLS) repair, accompanied by an altered intracellular NAD^+^/NADH ratio and excessive ROS accumulation. These findings demonstrate that combined suppression of DNA repair and metabolic homeostasis can jointly impair the survival resilience of tumor cells (59).

From the perspective of the tumor immune microenvironment, regulatory B cells with high LARS2 expression facilitate tumor immune escape by boosting mitochondrial NAD^+^ regeneration and oxidative metabolism. Targeting this pathway, such as Lars2 gene knockout or dietary leucine restriction, can impair the immunosuppressive function of regulatory B cells, strengthen anti-tumor immunity, and improve CRC prognosis. This provides a novel theoretical basis for the combined application of metabolic intervention and immunotherapy (13).

Furthermore, emerging research has explored the combination of NAD^+^ metabolic regulation and epigenetic modulation. Combined inhibition of NAMPT and CtBP transcription factors has achieved prominent synergistic anti-tumor effects in pancreatic cancer models (60). Although this research was not conducted in CRC, the dual-target concept linking metabolic remodeling and epigenetic regulation offers valuable insights for further verification in CRC research.

Overall, current preclinical evidence strongly supports the translational value of multi-modal combination regimens. Representative strategies include the co-administration of metabolic inhibitors and chemotherapeutics, combined targeting of metabolism and DNA repair, as well as integrated metabolic intervention and immune regulation. By simultaneously disrupting multiple vital vulnerabilities of tumor cells, these combinatorial schemes are expected to resolve the inherent limitations of single-pathway blockade, which is prone to being counteracted by tumor adaptive rewiring.

### 4.3 Limitations of current research and bottlenecks in clinical translation

Despite the theoretical potential of NAD^+^ metabolism-targeted interventions for CRC chemoresistance, their clinical translation faces multiple bottlenecks. Most mechanistic studies rely on conventional cell line models, which cannot recapitulate the spatiotemporal heterogeneity, stromal interactions, and dynamic immune microenvironment of patient-derived tumors. Although patient-derived cancer organoid (PDCO) models have been used to evaluate metabolic responses to regimens like FOLFOX (61), their standardized culture, long-term passaging stability, and high-throughput drug screening capacity need further optimization.

Second, *in vivo* dynamic monitoring technologies for intracellular NAD^+^ and NADH remain immature, limiting real-time assessment of metabolic flux changes after drug intervention. While label-free metabolic monitoring has been preliminarily achieved via WF ORI and two-photon NAD(P)H fluorescence lifetime imaging (FLIM) (61, 62), these approaches face resolution and throughput bottlenecks in deep-tissue *in vivo* detection, multi-time-point tracking, and clinical compatibility. The novel near-infrared fluorescent probe KC8 can target tumors intravenously and reflect p53 aberration-associated NAD(P)H changes (63), but its clinical translation requires large-scale validation.

Beyond these well-recognized translational hurdles, the fundamental research landscape of NAD^+^ metabolism in CRC chemoresistance also suffers from notable deficiencies in critical analysis and unresolved controversies. Most mechanistic studies prioritize clarifying the regulatory roles of core molecules and pathways in NAD^+^ metabolic reprogramming, yet neglect systematic reflection on the methodological limitations of experimental models—such as the poor recapitulation of tumor microenvironment heterogeneity by conventional cell lines and the inadequate long-term passaging stability of organoid models. Moreover, contradictory findings persist in the field: the bidirectional regulatory effects of NAD^+^ precursors on cancer stem cell survival vary across research contexts without a unified conclusion, and the functional orientation of SIRT family members remains inconsistent in different CRC subtypes, both of which underscore the underappreciated complexity of NAD^+^ metabolic regulation. More importantly, the current research fails to effectively bridge mechanistic insights with clinical application, leaving the clinical implementation of biomarker-guided therapy, patient stratification, and personalized treatment design largely ambiguous. While predictive biomarkers such as low NAPRT expression and high NAMPT expression have been identified to stratify CRC patients who may benefit from NAD^+^-targeted agents, their standardized detection methods, clinical validation in large cohorts, and integration into clinical trial design remain underexplored. Additionally, although several NAD^+^ pathway modulators have entered clinical trials, the lack of preclinical research that directly links molecular mechanistic data to clinical treatment efficacy has hindered the optimal design and patient selection for these trials. These unsolved scientific disputes, knowledge gaps, and the mechanistic-clinical disconnect not only hinder the in-depth dissection of NAD^+^-mediated chemoresistance mechanisms, but also create inherent barriers for the rational translation of NAD^+^-targeted strategies into clinical practice and the development of evidence-based intervention regimens.

Additionally, clinical translation of NAD^+^ modulators is limited by bioavailability. Compounds must cross the liver and resist gut microbiota “preemptive consumption” to reach tumor sites. Although oral NAD^+^ precursors like NMN elevate tissue NAD^+^ in murine models (64, 65), their human colon absorption efficiency is controversial, with species-specific transporter-dependent differences. Gut microbes metabolize NAD^+^ precursors (66), and degrade drugs, reducing efficacy (67). making delivery systems that bypass first-pass metabolism and microbial interference a critical bottleneck.

Most NAD^+^ metabolic inhibitors face off-target effects and dose-limiting toxicities. NAMPT inhibitor trials report gastrointestinal distress and hematologic adverse events (e.g., thrombocytopenia, neutropenia) (68). Tconstraining dosing and tolerability. SIRT family members have context-dependent dual functions (e.g., SIRT1 suppresses β-catenin signaling but inhibits p53-mediated apoptosis; SIRT3 promotes chemoresistance via SOD2/PGC-1α) (7), increasing clinical efficacy uncertainty. Validated biomarkers for patient stratification are lacking.

While NAPRT deficiency predicts NAMPT inhibitor sensitivity, its CRC incidence, detection standardization, and companion diagnostic development are in early stages. In summary, advancing this field requires more physiologically relevant models, high-precision dynamic monitoring tools, tissue-specific delivery strategies, and multi-dimensional omics-based precision subtyping.

## 5 Future directions of NAD^+^ metabolism-CRC chemoresistance research empowered by emerging technologies

### 5.1 Multi-omics integration and AI-driven discovery of chemoresistance biomarkers

In recent years, the integration of multi-omics technologies and artificial intelligence (AI) is reshaping the research paradigm for CRC chemoresistance mechanisms, exhibiting tremendous potential especially in dissecting pathways mediated by NAD^+^ metabolic reprogramming. By integrating genomic, transcriptomic, proteomic, and metabolomic datasets, researchers can construct dynamic maps of the NAD^+^ synthesis, catabolism, and regeneration networks within tumor cells, thereby identifying key nodes that drive chemoresistance (69). For instance, in CRC, dysregulated expression of NAD^+^-dependent enzymes—such as the PARP and SIRT families and CD38—is frequently accompanied by remodeling of glycolysis or the mitochondrial respiratory chain; these alterations can be precisely captured through joint multi-omics analysis and correlated with clinical chemoresistance phenotypes.

Most current AI models function as “black-box” tools that predict patient response probabilities but fail to reveal biological mechanisms or actionable metabolic targets. To address this translational gap, explainable AI (XAI) has emerged as a promising alternative. Attention-based multiple instance learning (MIL) can visualize key histopathological regions including fibrotic zones and immune-excluded architectures, converting algorithmic results into pathologist-interpretable evidence (70). Similarly, SHAP enables machine learning-based risk signatures to predict prognosis and quantify gene contributions such as RCN3 expression in cancer-associated fibroblasts, facilitating targeted therapeutic strategies (71).

In the context of NAD^+^ metabolism, explainability is particularly critical. Since NAD^+^/NADH imbalance often involves coordinated perturbations across multiple metabolic modules—including the pentose phosphate pathway, serine-glycine flux, and mitochondrial electron transport—traditional statistical models struggle to disentangle causal networks. Deep neural networks combined with graph neural networks (GNNs) can not only extract nonlinear features from high-dimensional heterogeneous data to predict drug sensitivity but also reveal, via node importance ranking or pathway activation heatmaps, which enzymatic hubs (e.g., NAMPT or NMNAT) are regulated by non-coding RNAs or epigenetic modifications to become resistance drivers (72). This mechanistic transparency allows clinical teams to prioritize the most “druggable” metabolic targets rather than rely blindly on probabilistic scores.

For example, evidence has shown that in oxaliplatin-resistant CRC cells, overactivation of PARP leads to downregulation of SIRT1 and increased glycolytic flux, while restoration of SIRT1 expression can reverse the drug-resistant phenotype (8). Combined with multi-omics data, AI models can identify key nodes in this pathway that are regulated by non-coding RNAs or affected by epigenetic modifications (such as the methylation status of the NAMPT promoter), and visualize their spatial distribution in the tumor microenvironment through graph neural networks, thereby matching potential beneficiaries of specific inhibitors (e.g., FK866) (56). Notably, when integrating transcriptomic and metabolomic data, deep learning models have been able to reveal how variations in the intronic region of the RHPN2 locus affect CRC cell proliferation by altering sucrose metabolism, and such mechanism-driven discoveries can be directly translated into wet-lab validation targets for CRISPR editing or small-molecule intervention (73).

Although most existing studies still focus on glucose or lipid metabolism (74), systematically embedding the NAD^+^ metabolic module into multi-omics frameworks—augmented by transfer learning strategies—holds promise for extracting more universal chemoresistance biomarkers from pan-cancer datasets. Future challenges lie in standardizing data pipelines, enhancing model interpretability (e.g., generating mechanism-driven hypotheses for wet-lab validation), and conducting prospective validation in independent clinical cohorts. Future directions should focus on prospective cohort validation to promote the transformation of AI models from “black-box prediction” to “mechanism-driven decision-making”. For instance, the AI-HOPE platform has demonstrated its ability to parse TCGA data through natural language queries, identify WNT/TGFβ pathway abnormalities, and recommend matched drugs with an accuracy of 0.98 (75). If the NAD^+^ metabolic pathway is incorporated into its knowledge graph, clinicians can directly input “mCRC patients with low SIRT1 expression and active glycolysis” to obtain personalized treatment recommendations. Standardized data pipelines, cross-center model transfer, and interpretable heatmap output will be key steps for achieving bedside deployment (76). Only in this way can AI-enabled multi-omics integration surpass the pan-cancer universal model in CRC precision treatment and be implemented as personalized and actionable metabolic intervention pathways. Only when AI not only “predicts accurately” but also “explains clearly” can its full clinical translation value in personalized oncology be realized (77).

### 5.2 Application prospects of organoid models and nanodelivery systems

The synergistic advancement of organoid models and nanodelivery technologies has provided a highly biomimetic, precisely controllable experimental and therapeutic platform for reversing CRC chemoresistance by targeting the NAD^+^ metabolic pathway. Patient-derived CRC organoids preserve the genetic background, metabolic characteristics, and drug response heterogeneity of primary tumors, rendering them an ideal *in vitro* system for evaluating NAD^+^ metabolism-targeted intervention strategies. Studies have shown that by administering NAD^+^ precursors (e.g., NMN or NR) or inhibitors (e.g., FK866) in organoid culture systems, it is possible to dynamically monitor cell viability, NAD^+^ pool fluctuations, and downstream acetylation/methylation epigenetic alterations, thereby screening personalized response regimens (78). Such models have been successfully applied to predict the resensitization potential of patients with oxaliplatin or 5-FU resistance, with a prediction accuracy of over 80% (78).

Meanwhile, nanodelivery systems offer an engineered solution to overcome the pharmacokinetic limitations of NAD^+^-related molecules. Carriers such as liposomes, polymeric micelles, or metal-organic frameworks enable tumor-targeted enrichment of NAD^+^ modulators, reduce systemic toxicity, and enhance intracellular delivery efficiency via pH- or enzyme-responsive release mechanisms (79). The latest advances include the development of bifunctional nanoparticles co-loaded with NAMPT inhibitors and chemotherapeutic agents, which not only restore NAD^+^ homeostasis but also significantly delay the emergence of drug-resistant clones in CRC mouse models (80). Nanoplatforms can also be integrated with imaging probes to achieve real-time therapeutic efficacy feedback during treatment.

Current bottlenecks include the insufficient complexity of the organoid microenvironment, the scalability and stability of nanocarrier mass production, and the evaluation of long-term *in vivo* biocompatibility. Breaking through these limitations will accelerate the translation of NAD^+^ metabolism-targeted interventions from the laboratory to clinical practice.

Future clinical interventions can utilize the PDO platform to real-time monitor fluctuations in the NAD^+^ pool and alterations in downstream acetylation/methylation modifications, so as to accordingly regulate the drug combination (e.g., NAMPT inhibitor combined with conventional chemotherapeutics, or PARP inhibitor plus 5-FU) and release kinetics of nanoformulations. Meanwhile, the integration of imaging probes enables visualized feedback on tumor metabolic responses during treatment, which further optimizes the administration schedule and dosage at the individual patient level.

Current bottlenecks, including insufficient complexity of the organoid microenvironment and unstable performance of nanocarriers in large-scale production, are being addressed via the adoption of matrix co-culture systems and engineered protein corona regulation technologies. With the maturation of the above technologies, NAD^+^ metabolic reprogramming is poised to advance from mechanistic exploration to a clinical pathway for precise drug resistance reversal in CRC, wherein PDOs serve as the navigation tool and nanosystems act as the execution unit.

### 5.3 Technological breakthroughs in dynamic metabolic imaging and real-time monitoring

Advances in dynamic metabolic imaging technologies are driving the study of NAD^+^ metabolism in CRC chemoresistance from static characterization to spatiotemporally resolved functional monitoring, providing unprecedented visualization tools for elucidating the relationship between metabolic heterogeneity and therapeutic resistance. FLIM enables the discrimination of the ratio of free to protein-bound coenzymes by detecting changes in the autofluorescence lifetime of NAD(P)H, indirectly reflecting cellular redox status and metabolic flux without exogenous labeling. Recent studies have utilized FLIM to identify chemoresistant subpopulations with high NADH binding fractions in CRC tissue sections; their spatial distribution is highly coincident with tumor invasive fronts, indicating the critical role of the local metabolic microenvironment in the selection of chemoresistant clones (81).

In addition, hyperpolarized ¹³C-MRI technology allows *in vivo* tracking of the conversion rate of [1-¹³C]nicotinamide or [¹³C]tryptophan to NAD^+^ via the kynurenine pathway, thereby quantifying the dynamic changes in NAD^+^ regenerative capacity under different therapeutic interventions (82). This method has been successfully applied in pancreatic cancer models to predict the differential therapeutic efficacy of radiotherapy and hypoxia-activated prodrugs (82), and its principles are translatable to CRC chemoresistance research. Emerging Raman spectroscopic imaging and mass cytometry have further improved single-cell resolution, enabling researchers to synchronously map three-dimensional profiles of NAD^+^ concentration, related enzyme activities, and drug distribution in organoids or patient biopsy samples.

The clinical translation of these technologies still faces challenges such as signal-to-noise ratio optimization, scanning speed enhancement, and cost control. However, with the integration of miniaturized probes, compressed sensing algorithms, and artificial intelligence image analysis modules, the aforementioned imaging systems are expected to be embedded into endoscopic platforms or intraoperative navigation devices in the future, enabling a “see-and-treat” closed-loop treatment model—for example, real-time identification of metabolically abnormal lesions during laparoscopic surgery and synchronous release of sustained-release NAD^+^-depleting nanoparticles. This pathway not only focuses on the unique spatial architecture of the metabolic microenvironment and molecular interaction network of CRC but also emphasizes the realization of technological integration within the existing clinical operation framework, thereby accelerating the translation from mechanistic insight to bedside application.

**Figure 3:**
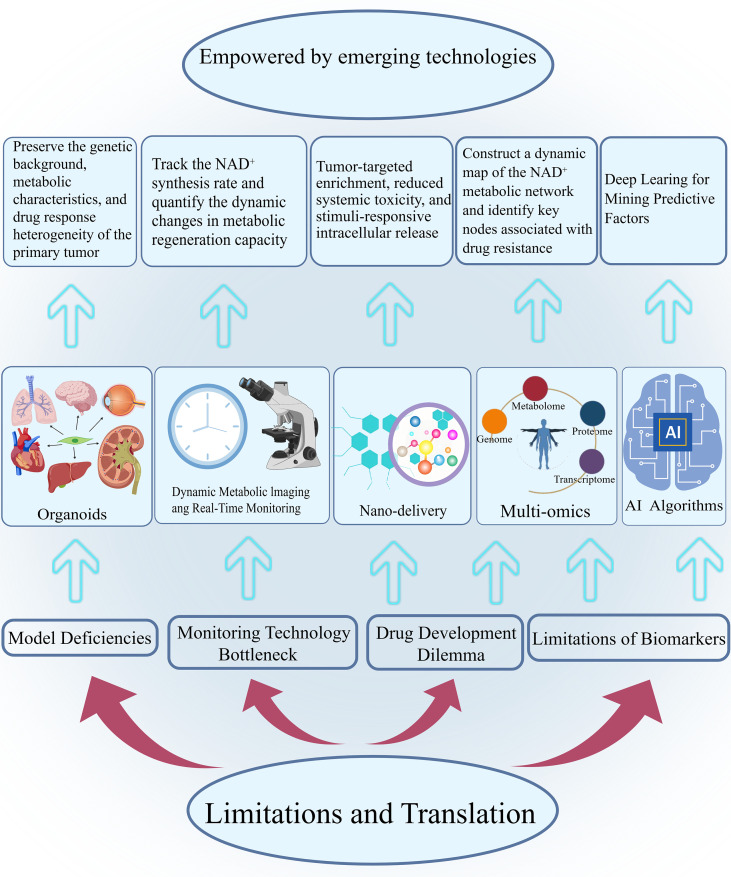
Emerging technologies-enabled advances in NAD^+^ metabolism research for CRC chemoresistance. This schematic illustrates how emerging technologies address key limitations in translating NAD^+^ metabolism research into clinical practice for CRC chemoresistance. Organoid models preserve tumor genetic and metabolic heterogeneity, while dynamic metabolic imaging enables real-time tracking of NAD^+^ synthesis and regeneration. Nano-delivery systems enhance tumor targeting and reduce systemic toxicity, multi-omics approaches construct dynamic metabolic networks to identify resistance-associated nodes, and AI algorithms mine predictive factors for patient stratification. Together, these technologies bridge critical gaps in model deficiencies, monitoring bottlenecks, drug development dilemmas, and biomarker limitations, accelerating the translation of NAD^+^-targeted strategies.

To visually summarize the above directions, **Figure 3** illustrates how emerging technologies to advance NAD^+^ metabolism research in CRC chemoresistance.

## 6 Conclusion

As a core regulatory mechanism underlying CRC chemoresistance, NAD^+^ metabolic reprogramming endows tumor cells with survival advantages through multidimensional remodeling of energy metabolism, epigenetics, and the immune microenvironment. Targeted interventions against this process exhibit broad-spectrum and precise anticancer potential. However, bottlenecks including the complex metabolic network induced by tumor heterogeneity, the lack of dynamic monitoring technologies, and insufficient tissue specificity of drugs have hindered the progress of clinical translation. Breakthroughs in this research field are crucial for improving the predicament of CRC treatment and thus deserve great attention from both the academic and clinical communities.

In the future, it is necessary to rely on emerging technologies such as multi-omics integration, organoid models, nanodelivery systems, and dynamic metabolic imaging to dissect metabolic heterogeneity, develop tissue-specific modulators and combination therapies, and advance from mechanistic research to personalized chemoresistance intervention, thereby bringing new therapeutic hope to patients with CRC. For a broader cross-cancer perspective, **Supplementary Table 1** summarizes the roles of the NAD⁺ metabolic pathway in multiple cancers (83–103).
