# Supplementary material for: The role of NAD+ metabolic reprogramming in colorectal cancer chemoresistance: mechanistic insights, clinical translation challenges and opportunities
Source: Front Oncol. 2026 May 28;16:1784749. doi: 10.3389/fonc.2026.1784749 (PMC13253425; doi:10.3389/fonc.2026.1784749)
Supplement: Supplementary file 1 [file Table1.docx]

**Supplement Table 1**. Roles of NAD⁺ metabolic pathway in multiple cancers.

| **Cancer Type** | **Abnormality** | **Biological Effect** | **Therapeutic Targets** | **Research Model** | **References** |
| --- | --- | --- | --- | --- | --- |
| CRC | NAD⁺ metabolism gene expression disorder; high NAD⁺ level | Prognosis correlation; stemness maintenance; chemoresistance | NAMPT, SIRT1 | CRC cells, patient tissues, mouse xenografts | (22,90,91) |
| Prostate Cancer | NAPRT deficiency; SIRT4 downregulation | NAMPT pathway dependence; glutamine metabolism; metastasis | NAMPT, NNMT | 22Rv1 cells, CRPC-SCL mice, clinical samples | (92,93) |
| Multiple Myeloma | Macrophage NAMPT overexpression; high CD38 | Immunosuppression; chemoresistance; NAD⁺ depletion | NAMPT, CD38 | Macrophages, MM cells, scRNA-seq | (92,94,95) |
| Pancreatic Cancer | BICC1/IDO1 upregulation; CtBP overactivation | NAD⁺ synthesis; stemness; transcription regulation | NAMPT, CtBP, IDO1 | PDAC cells, xenografts, organoids | (67,93,96) |
| Lung Cancer | NADK mutation; lactic acid-induced CD38 | Chemotherapy resistance; EMT | NADK, SIRT1 | Kras mice, LLC/GLC-82 cells, patient tissues | (94,97–99) |
| Glioma | IDH1 mutation; NMNAT1/2 upregulation | NAD(P) depletion; anti-apoptosis; redox imbalance | NMNAT, NRF2, IDH1 | Glioma cells, Drosophila, TCGA/CGGA | (100–102) |
| Ovarian Cancer | NAD⁺ salvage enhancement; PHGDH downregulation | Platinum resistance; migration; stemness | NAMPT, DNMT, PARP | A2780, resistant lines, PDX | (103–105) |
| Liver Cancer | NAD⁺ metabolism heterogeneity; low NAPRT/NAMPT | Poor prognosis; cachexia | NRK2, NAD⁺ precursors | TCGA-LIHC, patient blood, cachexia mice | (106–108) |
| Breast Cancer (Triple-Negative) | NAMPT upregulation; SIRT1-p66Shc disorder | EMT; metastasis; endocrine resistance | NAMPT, SIRT1 | TNBC PDX, MCF-7/LTED, metastasis models | (109,110) |
| Rhabdomyosarcoma | NAPRT silencing | Synthetic lethality to NAMPTi | NAMPT | RMS cells, NAPRT KO/OE, orthotopic mice | (111,112) |
